# The ASLEEP Intervention for Insomnia Symptoms in Mid-Life and Older Adults supported by the PROTECT Norge Platform: Single-Arm, Multimethod Feasibility and Acceptability Study

**DOI:** 10.2196/86591

**Published:** 2026-07-30

**Authors:** Jon Arild Aakre, Ingelin Testad, Martha Therese Gjestsen, Lise Birgitte Holteng Austbø, Clive Ballard, Bjørn Bjorvatn

**Affiliations:** 1 Department of Clinical Medicine, University of Bergen, Laboratory Building, 7th floor, Haukeland University Hospital, Jonas Lies vei 91 B, Bergen, Vestland, 5009, Norway; 2 Centre for Age-Related Medicine - SESAM, Stavanger University Hospital, Stavanger, Rogaland, Norway; 3 Department of Health and Community Sciences, Faculty of Health and Life Sciences, University of Exeter, Exeter, England, United Kingdom; 4 Department of Clinical Biosciences, Faculty of Health and Life Sciences, University of Exeter, Exeter, England, United Kingdom; 5 Department of Global Public Health and Primary Care, University of Bergen, Bergen, Vestland, Norway; 6 Norwegian Competence Center for Sleep Disorders, Haukeland University Hospital, Bergen, Vestland, Norway

**Keywords:** insomnia, middle-aged and older adults, eHealth intervention, digitally delivered cognitive behavioral therapy for insomnia, eCBT-I, feasibility study, acceptability, multimethod, sleep health

## Abstract

**Background:**

Sleep is essential for health, yet a large proportion of the population reports sleep complaints. The ASLEEP (Preventing and Treating Insomnia Symptoms in Mid-Life and Older Adults) intervention is conceptualized as an eCBT-I (digitally delivered cognitive behavioral therapy for insomnia) intervention aiming to prevent and treat insomnia in individuals aged 50 years and over through a novel, tiered approach. The first step, a basic course delivered via iPad through a dedicated app, includes educational videos grounded in sleep education and sleep hygiene advice, a 7-day digital sleep diary, and a postcourse digital sleep report providing personalized feedback on sleep efficiency via email.

**Objective:**

The objective of this study was to assess the feasibility and acceptability of the ASLEEP basic course among adults aged 50 years and over, including participant engagement, acceptability of the course content, and delivery of study activities, through PROTECT Norge (Platform for Research Online to Investigate Genetics and Cognition in Ageing Norge), an online research infrastructure.

**Methods:**

A single-arm multimethod feasibility and acceptability study was conducted as a nested study within PROTECT Norge. Data comprised preintervention questionnaires on sleep and mental health, intervention compliance tracking, and 2 postintervention focus group interviews. Questionnaire and compliance data were analyzed descriptively, and focus group data were analyzed using systematic text condensation.

**Results:**

Of 150 invited individuals, 18 expressed interest. Ten participants were included in the study, all of whom completed the intervention and subsequently took part in 2 focus group interviews, with 5 participants in each group. Participants included 6 women and 4 men aged 54 to 78 years. Preintervention data showed that 7 participants reported subthreshold insomnia and 3 reported no insomnia. Sleep diary compliance during the ASLEEP basic course was 99.3%. The systematic text condensation analysis revealed three main categories: (1) motivation for participation in the ASLEEP study, (2) benefits of the ASLEEP intervention, and (3) promoting and hindering factors of delivering a fully digitalized intervention.

**Conclusions:**

The ASLEEP basic course was well-received and provided participants with a new understanding of sleep through increased knowledge and awareness. Technological insecurity was experienced by some of the participants, but it was mitigated by technical support. Improving app stability and expanding device compatibility could enhance accessibility and optimize ASLEEP’s future delivery. The PROTECT Norge platform demonstrated good feasibility in recruitment, participant management, and compliance monitoring, supporting the successful delivery of the study.

## Introduction

Sufficient sleep is critical for optimal daytime functioning, overall health, and quality of life. In terms of sleep duration, this typically entails obtaining between 6 and 9 hours of sleep per night [1]. With age, sleep becomes lighter and more fragmented, with reductions in slow-wave and rapid eye movement (REM) sleep, longer sleep onset latency, increased awakenings [2], and a circadian shift toward morningness [3]. Combined with declining health, greater medication use, reduced physical activity, and heightened mental health concerns, older adults are a high-risk group for insomnia [4]—a condition characterized by difficulty falling or staying asleep, or waking too early, causing significant daytime impairment [5]. Insomnia is strongly associated with poorer mental health outcomes, while being sleep deprived is linked to adverse physical health outcomes such as cardiovascular and neurodegenerative diseases, obesity, hypertension, and increased mortality—supporting calls to promote sleep as a fundamental pillar of health, on par with nutrition and physical activity [6].

Insomnia prevalence in Europe has previously ranged from 5.8% to 34.8% among adults across various countries [7]. A recent Norwegian study suggests an increasing prevalence, with rates reaching 25%, placing Norway among the countries with the highest reported levels [8], though the methodology may have limited differentiation between insomnia and symptoms of other conditions in this specific study. Older adults represent a high-risk group for insomnia, an issue that is becoming increasingly significant as the number of older adults, particularly in rural areas, is projected to rise in Norway [9] and the rest of Europe as well [10]. For chronic insomnia, defined as lasting 3 months or more, cognitive behavioral therapy for insomnia (CBT-I) is the gold standard treatment, traditionally delivered by a specialist over several and individually structured sessions based on patients’ needs [11]. Insomnia is underdiagnosed and undertreated, and there are significant challenges in making adequate treatment widely accessible, partly because traditionally delivered CBT-I is resource demanding and a lack of qualified personnel to deliver treatment [7]. Thus, only 20% of insomnia cases in the general population are believed to receive adequate treatment [12].

As digital technologies become increasingly integrated into everyday life across age groups and societal sectors, population-level digital literacy is concurrently increasing. In Norway, 80% of adults aged 16 to 74 years possess basic digital skills [13], indicating a high level of digital readiness that can be leveraged to improve access to health care through new digital delivery channels. However, limited digital skills among older adults raise concerns about their inclusion and ability to participate in the ongoing digital transformation [14]. In the context of eHealth, in addition to age, social determinants such as education, income, and social support independently contribute to individuals’ uptake of and ability to fully engage with eHealth services and resources [15]. Thus, addressing these factors is critical to ensuring the equitable implementation of eHealth initiatives. For eHealth interventions to be effective in aging populations, it is important to ensure accessibility, offer training and support, and design user-friendly, needs-based content [16,17], while incorporating psychological behavior change theories, which in general enhance behavior change and can further improve their impact [18]. eHealth-based psychosocial interventions have been shown to significantly reduce insomnia severity and improve sleep quality in both clinical and subclinical populations [19]. Educational approaches, a core component of CBT-I, which includes psychoeducation, have also been proposed as viable options to address the increasing public health concerns regarding sleep complaints in the general population [20,21].

Although there is now a substantial body of evidence demonstrating the efficacy of eCBT-I (digitally delivered cognitive behavioral therapy for insomnia) [19], access to evidence-based interventions, such as SLEEPIO [22] and Somryst [23], is not freely available in many countries, including Norway, reflecting broader challenges in integrating such digital sleep interventions into care pathways and ensuring long-term availability. Building on a CBT-I self-help book shown to be more effective than stand-alone sleep hygiene advice [24], the ASLEEP (Preventing and Treating Insomnia Symptoms in Mid-Life and Older Adults) intervention is conceptualized as an eCBT-I program aiming to prevent and treat insomnia in individuals aged 50 years and over through a novel tiered approach. A basic course promoting healthy sleep through psychoeducation has been developed, with an advanced course in planning to provide targeted CBT-I techniques for those needing additional support within the PROTECT Norge (Platform for Research Online to Investigate Genetics and Cognition in Ageing Norge) cohort. PROTECT Norge is an online platform dedicated to research on the aging brain and involves over 5000 research-willing Norwegian adults aged 50 years and over who complete study activities via a dedicated website, and the platform itself holds nested study functionalities [25]. Although the ASLEEP intervention is grounded in evidence-based principles, it remains unclear whether it can effectively attract and engage a diverse group of community-dwelling adults aged 50 years and over, both with and without insomnia, within the PROTECT Norge cohort. In line with recommendations from the Medical Research Council framework for developing and evaluating complex interventions [26], the aim of this study was to assess the acceptability of the intervention’s content among this target population, as well as the feasibility of delivering study activities through the existing PROTECT Norge infrastructure. To support the study’s relevance, user-friendliness, and accessibility, patient and public involvement and engagement (PPIE) was incorporated alongside the research design.

## Methods

### Study Design

Guided by the Medical Research Council framework [26] and the concept of information power [27], this study employed a single-arm feasibility and acceptability design, using a multimethod approach with preintervention and postintervention assessments, respectively, collected through questionnaires and focus group interviews, followed by both quantitative and qualitative analyses. Reporting of the qualitative component of this study was guided by the SRQR (Standards for Reporting Qualitative Research; Checklist 1) [28].

The acceptability of the intervention was the primary outcome of interest. Preintervention testing and sleep diary compliance were used to describe the characteristics of the participants and to provide an indication of their engagement with the intervention.

### Intervention

The ASLEEP intervention’s basic course is an evidence-based and theory-driven eHealth intervention designed to promote healthy sleep within the PROTECT Norge population. The basic course represents tier 1 of the planned ASLEEP stepped-care intervention, with an advanced course representing tier 2, and is only intended for individuals with insomnia symptoms, requiring more intensive eCBT-I treatment [29]. The present feasibility and acceptability study evaluated only the basic course. The intervention combines digital education through video modules, keeping a sleep diary, and giving tailored sleep feedback based on sleep diary data through a sleep report, delivered digitally to participants’ emails. Table 1 outlines the content of the videos. All but the sleep diary instruction—delivered solely by JAA—feature dialogues between JAA and BB discussing specific topics, supplemented with multimedia content to engage and visually emphasize important and complex points.

**Table 1.** ASLEEP^a^ intervention video modules, including module length, title, and educational content.

Video/length	Duration (min)	Name	Description
Sleep diary instruction	17:17	Instruction to digital sleep diary	How to use the digital sleep diary and general misconceptions related to completing a sleep diary
Video module 1	20:03	What is sleep and how it is regulated	Provides an overview of sleep, its bodily functions, and the sleep-regulating mechanisms
Video module 2	18:10	Assessing poor sleep	Causes and the diagnosis of sleep problems and a general overview of the various sleep-wake disorders
Video module 3	30:10	Treatment of insomnia	Focuses on the management of insomnia, including definitions, prevalence, and treatment strategies for both short-term and chronic insomnia, as well as principles of sleep regulation. Additionally, Professor Bjorvatn gives sleep hygiene advice, discussing ways to maintain sufficient sleep, support a healthy circadian rhythm, and minimize evening and nighttime arousal through habits and behavioral practices

a ASLEEP: Preventing and Treating Insomnia Symptoms in Mid-Life and Older Adults.

The sleep report provides participants with personalized feedback on their sleep based on sleep efficiency, which is calculated from sleep diary data collected over 7 days, delivered postintervention via email. Participants are instructed to complete the digital sleep diary twice daily: a morning entry before noon, ideally immediately after waking, and an evening entry before 11:59 PM, preferably just before going to bed. The report explains what sleep efficiency is, how it is calculated, its interpretation and practical significance, and also includes a disclaimer cautioning against using it for self-diagnosis of sleep-wake disorders.

Groupwise, in-person training is provided to participants to familiarize them with the iPad and the app and to give an introduction to filling out the sleep diary prior to the start of the intervention.

### Setting

The ASLEEP intervention was delivered in a hybrid format, combining both in-person and digital components. The initial informational meeting and final focus group interviews were conducted at the Centre for Age-Related Medicine (SESAM), Stavanger University Hospital. All other study activities were completed remotely, supported by the PROTECT Norge infrastructure and the Berntsen app (Innocom).

The PROTECT Norge infrastructure facilitated participant registration, digital informed consent, preintervention testing (via baseline questionnaires), and compliance tracking. Participants accessed their personal PROTECT Norge study dashboard to complete required activities and log compliance. The Berntsen app, preinstalled on study-provided iPads, delivered the core intervention components—including the digital sleep diary, educational video modules, and personalized sleep reports.

Participant activity across both digital systems was supported by a combination of automated reminders and personalized follow-up via email, SMS, and phone. Study activity monitoring and participant support were conducted by trained staff at the Centre for Age-Related Medicine (SESAM) using the PROTECT Norge admin dashboard, which provided real-time access to participants’ study progression through a downloadable whitelist report. This enabled timely identification of participants who required reminders or technical assistance. Dedicated technical support was made available daily from 08:00 AM to 10:00 PM, ensuring that participants could receive help as needed throughout the study period.

### Participants and Recruitment

#### Inclusion and Exclusion Criteria and Screening Process

In line with the inclusion and exclusion criteria in PROTECT Norge, the target group for the intervention is cognitively healthy individuals aged 50 years and over, residing in Norway, who have a good understanding of the Norwegian language and access to a computer or tablet device with internet connectivity. The PROTECT Norge cohort has primarily been recruited through public outreach activities and social media, particularly Facebook [25].

Potential participants were identified by screening the PROTECT Norge cohort [25]. Specifically, active PROTECT Norge participants who had provided consent to be contacted for future research (n=4296) were screened for eligibility based on their postal code to determine residency in Stavanger municipality. This prescreening process identified 508 eligible individuals meeting criteria for participation in the ASLEEP study.

#### Recruitment Process

A nonrandom sequential invitation strategy was employed in the screened PROTECT Norge cohort. Sequential invitations were sent via the official PROTECT Norge email between March 7 and 11, 2024, starting from the top of the list of 508 eligible individuals. Invitations were sent until the recruitment target of 10 participants was reached; at this point, 150 individuals had received an invitation. A total of 18 individuals responded showing interest in participating, of whom 8 were excluded either because of being unable to attend on-site study activities on specified dates—including postintervention focus group interviews—or because they responded after the recruitment has closed.

The recruitment followed a staged, first-come, first-served approach with eligibility and availability assessed by the first author (JAA). Interested individuals were contacted via SMS, with follow-up calls offered as needed. Subsequent verbal or written confirmation of interest in the study resulted in the ASLEEP study being activated on the participant’s PROTECT Norge account, where candidates could complete enrollment by registering and providing consent, in line with the consent procedure described in detail in a previous publication [25].

The focus group component was embedded in the ASLEEP intervention recruitment pathway rather than being recruited separately. Therefore, the focus group sample reflected criterion-based sampling, as participants had received the ASLEEP intervention and had confirmed their availability and willingness to attend the postintervention focus groups, enabling feedback from participants with direct experience of the intervention.

#### Sample Size

The sample size for the focus group interviews was guided by the concept of information power [27]. Ten participants were considered sufficient prior to data collection, at which point recruitment was stopped. A list was retained of interested individuals who were excluded due to unavailability on the scheduled study dates or who expressed interest after the recruitment stopped, allowing them to be invited to a subsequent intervention round and focus group, should the initial sample provide insufficient information power.

### Data Collection

#### Quantitative Data Collection

Demographic information, including age, sex, ethnicity, marital status, education, and employment, was previously collected as part of the PROTECT Norge study. A composite questionnaire was administered at baseline to assess symptoms of insomnia, depression, and anxiety. It included the Insomnia Severity Index [30], the Patient Health Questionnaire [31] (PHQ-9), and the Generalized Anxiety Disorder scale [32] (GAD-7). A sleep variable was incorporated into the composite questionnaire to specifically capture the duration of participants’ sleep problems: “How long have you experienced sleep problems?” with the following response categories: “I have not experienced sleep problems,” “Less than a month,” “One to two months,” and “Three months or more.” This allowed for the distinction between acute and chronic insomnia symptoms.

#### Qualitative Data Collection

Two exploratory focus group interviews were conducted, each comprising 5 individuals who had participated in the ASLEEP intervention, yielding a total of 10 participants. The purpose was to explore participants’ experiences and perceptions of the intervention, including its eHealth delivery platform and specific components such as the video modules, digital sleep diary, and personalized sleep report. A semistructured interview guide (Textbox 1) was used to facilitate discussion, consisting of 9 core questions supported by optional prompts. The guide served as a flexible framework, allowing moderators to pursue relevant follow-up questions and elicit detailed accounts of participant experiences, including reflections on specific incidents or aspects of the intervention. All moderators had prior experiences with conducting and moderating focus groups. Each session was moderated by JAA, with IT and LBHA acting as co-moderators for 1 interview each, responsible for note-taking, managing the interview guide and time, and contributing additional questions as needed. As part of his PhD work, JAA led the development and evaluation of ASLEEP and contributed to coordinating PROTECT Norge, giving him detailed contextual knowledge and a potential insider-outsider position. While this familiarity supported contextual understanding, it also introduced a risk of assumed understanding. To reduce this risk, JAA summarized key points during the interviews and invited participants to clarify or elaborate before moving to new topics. The interviews were audio-recorded and transcribed verbatim by external professional transcribers, with participants being pseudonymized during transcription. Subsequently, JAA reviewed the transcripts against the recordings to ensure transcription accuracy and support interpretation of the material in light of the interview context.

Textbox 1. **Semistructured focus group interview guide.**

1. What was your motivation for enrolling in the course?

2. Were there specific elements of the course that you appreciated?

2.1 Did you learn anything new during the course?

2.2 What aspects of the course did you find relevant?

3. What previous experience did you have using technology?

4. How did you find using the iPad to participate in the course?

4.1 You had to connect the iPad to the Berntsen app on your home network. How did that work?

4.2 Did the technology function as it should?

4.3 Did you experience any technical problems during the course?

5. How was it using the Berntsen app?

5.1 Was it easy to use and navigate?

5.2 Did you easily find what you were looking for?

6. What did you think of the videos?

6.1 Was the information understandable?

6.2 Was the information engaging?

6.3 Were videos a suitable way to receive information?

6.4 Was the length of the videos appropriate?

6.5 Were there elements in the videos that were particularly useful?

6.6 Were there elements in the videos that were not particularly relevant?

6.7 Was there anything that could have been done differently?

7. What did you think about completing a sleep diary?

7.1 Did you find the digital solution a suitable way to complete a sleep diary?

7.2 Did you find the digital solution for completing the sleep diary easy to use and navigate?

7.3 Were the reminders to complete the sleep diary in the Berntsen app useful?

7.4 Did you watch the introduction to the sleep diary video?

8. What do you think of the sleep report?

8.1 Was the information understandable?

8.2 Was the sleep report a suitable way to receive information about your sleep?

8.3 Was there anything you felt was missing from the sleep report?

9. Was there anything you felt was missing or should have been given more focus in the course?

**Figure 1.**
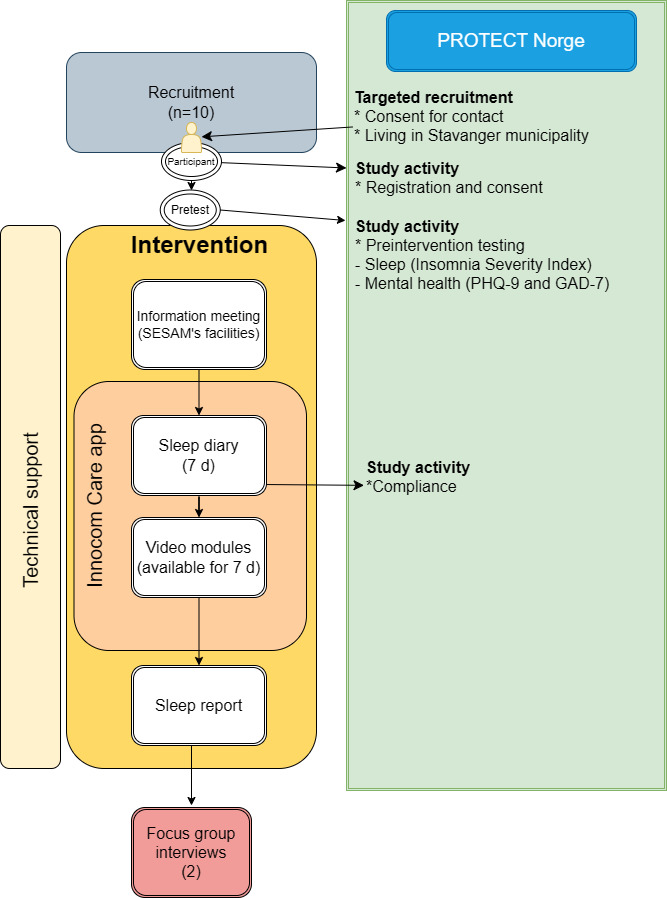
Key elements and timeline of the ASLEEP (Preventing and Treating Insomnia Symptoms in Mid-Life and Older Adults) study. GAD-7: Generalized Anxiety Disorder 7-item scale; PHQ-9: Patient Health Questionnaire-9; PROTECT Norge: Platform for Research Online to Investigate Genetics and Cognition in Ageing Norge; SESAM: Centre for Age-Related Medicine.

Figure 1 illustrates the targeted recruitment of participants who had provided consent to be contacted and resided in Stavanger municipality; the processes of registration, consent, preintervention testing, and compliance registration through the PROTECT Norge infrastructure; intervention delivery through an information meeting, a 7-day digital sleep diary, video modules available for 7 days, and a sleep report; technical support throughout the intervention; and 2 postintervention focus group interviews.

### Data Analysis

#### Quantitative Analysis

Descriptive analyses, including frequency distributions and the transformation of variables for categorical presentation, were conducted using IBM SPSS Statistics 26. Depression severity was categorized based on PHQ-9 scores: 0 to 4 (no depression), 5 to 9 (mild), 10 to 14 (moderate), 15 to 19 (moderately severe), and 20 to 27 (severe) [31]. Anxiety severity was categorized based on GAD-7 scores: 0 to 4 (no anxiety), 5 to 9 (mild), 10 to 14 (moderate), and 15 to 21 (severe) [33]. Insomnia severity was categorized based on Insomnia Severity Index sum scores: 0 to 7 (no insomnia), 8 to 14 (subthreshold insomnia), 15 to 21 (moderate insomnia), and 22 to 28 (severe insomnia) [30]. For both the PHQ-9 and GAD-7, scores ≥10 were used as thresholds for clinically significant symptoms of depression and anxiety, respectively, reflecting established cutoffs for moderate or greater symptom severity [34]. No inferential statistics were applied; thus, the results were limited to descriptive summaries of categorical severity levels across the sample.

#### Qualitative Analysis

Systematic text condensation (STC) was used to analyze the interview transcriptions. STC was chosen because it provides a highly structured and pragmatic analytic approach and is widely applied for descriptive cross-case analysis of participants’ experiences in medical qualitative research [35]. In addition, the researchers involved in the analysis had previous experience using this method. Before and during the analysis, active reflexive processes were used to identify and bracket preconceptions, including prior knowledge of ASLEEP and PROTECT Norge, expectations regarding the intervention components, and awareness of possible barriers to digital engagement among older adults. In line with STC, these preconceptions were revisited throughout the analytic process as both resources and potential sources of blind spots. Analytic reflexivity was supported through the collaborative analytic procedures described below.

The first step of the analysis involved gaining an overview of the transcribed material. Two researchers (JAA and IT) independently read the transcripts to identify preliminary themes. These themes were then discussed jointly and presented to ATHENA—a multidisciplinary health research group—which included, among others, coauthors MTG and IT, to incorporate broader analytical perspectives. In the second step, following discussion on the preliminary themes, JAA identified meaning units, which are text fragments from the transcript that contain meaningful information. The meaning units were coded and sorted to reflect the preliminary themes from the first step. The third step involved developing subgroups that captured the core content of each code group, forming condensates—artificial quotations closely related to the original expressions from the informants—and identifying illustrative quotes. Finally, the fourth step involved synthesizing the condensates into reconceptualized descriptions, also referred to as analytic text, which conveyed key aspects of the participants’ experiences. Steps 3 and 4 were primarily undertaken by JAA in close collaboration with IT through iterative discussions during the analytic process. An audit trail documenting key analytic decisions was maintained throughout the analysis.

### PPIE Through WiseAge

PPIE was integrated alongside the research design to help ensure that the study was relevant and accessible to its intended users. PPIE activities are facilitated through WiseAge, a platform for user involvement and societal engagement that was established by SESAM at Stavanger University Hospital. WiseAge has an implemented infrastructure to ensure patient and public involvement across all research activities at SESAM. This structure includes a user panel that meets regularly, where researchers present their research proposals or project plans for discussion. During these meetings, researchers are asked to answer questions and receive general feedback from panel members as a means of ensuring the relevance of planned research. In addition, 1 or 2 WiseAge members are typically assigned to each project and follow it through to completion to secure user perspectives throughout all phases of research. Continuous dialogues between researchers and user representatives are maintained through regular meetings and email correspondence. PPIE in this study is reported according to the GRIPP2 (Guidance for Reporting Involvement of Patients and the Public, Version 2) short-form checklist [36].

### Ethical Considerations

Participants in this study were recruited from the PROTECT Norge study [25], which holds ethical approval (reference number 2019/478). This study was assessed by a Regional Committee for Medical and Health Research Ethics and determined not to require research ethics committee approval. Informed consent was obtained from all participants through a validated web-based procedure. Data were pseudonymized prior to analysis and stored in accordance with the General Data Protection Regulation and Stavanger University Hospital’s internal data security policy. No financial or material compensation was provided for participation in this study.

## Results

### Outcomes of Patient and Public Involvement

The WiseAge user representatives contributed to refining the materials and procedures used in the ASLEEP feasibility study. Their input during early testing of the PROTECT Norge platform helped identify minor textual and usability issues, which were corrected before the feasibility study. Feedback on the intervention videos was also received, mainly concerning clarity and pacing, and informed small postproduction adjustments. These contributions supported the preparation of clear and accessible study materials tailored to the study’s target population, as well as a functional digital platform for participant use.

### Sociodemographic Characteristics of the Sample

Table 2 provides an overview of the ASLEEP study participants, all of whom completed the intervention and subsequently took part in the focus group interviews. Participants’ ages ranged from 54 to 78 years, with an average age of 65 (SD 8.8) years. The group included 6 women and 4 men, all of whom identified as being of Western ethnicity. The majority (7 out of 10) were married, while the remaining 3 were either divorced or widowed. In terms of education, 9 participants held a university degree, and 1 had completed secondary school. Employment status varied: half were retired, while the others were still working either full-time or part-time.

**Table 2.** Sociodemographic characteristics of the ASLEEP^a^ study participants^b^.

Demographic variables	Number of participants, n
Sex	
Male	4
Female	6
Ethnicity^c^	
Western European/North American/Oceanian (“Western”)	10
Other	0
Marital status	
Married	7
Widowed	1
Divorced	2
Education	
Secondary education	1
University, 4 years or more	6
PhD	1
University, less than 4 years	2
Employment	
Employed (full-time)	3
Employed (part-time)	2
Retired	5

a ASLEEP: Preventing and Treating Insomnia Symptoms in Mid-Life and Older Adults.

b Participants’ ages ranged from 54 to 78 years, with an average age of 65 (SD 8.8) years.

c Ethnicity was self-reported using the following predefined response options: Western European/North American/Oceanian (“Western”), Eastern European, East Asian, West Asian, Southern African, North African/Middle Eastern, Sámi, and Other ethnic origin. In this sample, all participants selected Western European/North American/Oceanian (“Western”); therefore, the remaining response options are presented collectively as “Other.”

### Preintervention Prevalence and Severity of Insomnia, Depression, and Anxiety

Table 3 summarizes the distribution of participants across different levels of insomnia severity and duration of sleep problems, current depression, and current anxiety, based on their respective scores from the ASLEEP composite questionnaire. The counts indicate how many participants fell into each category of insomnia severity, the duration of any experienced sleep problems, and whether they met the criteria for current depression or anxiety.

**Table 3.** Preintervention distribution of insomnia severity, duration of sleep problems, depression symptoms, and anxiety symptoms among ASLEEP^a^ study participants.

Measure and category	Count
Insomnia severity	
No insomnia	3
Subthreshold insomnia	7
Moderate insomnia	0
Severe insomnia	0
Duration of sleep problems	
I have not experienced sleep problems	1
Less than a month	0
1 to 2 months	0
3 months or more	9
Current depression (PHQ-9^b^≥10)	
No	9
Yes	1
Current anxiety (GAD-7^c^≥10)	
No	10
Yes	0

a ASLEEP: Preventing and Treating Insomnia Symptoms in Mid-Life and Older Adults.

b PHQ-9: Patient Health Questionnaire-9.

c GAD-7: Generalized Anxiety Disorder 7-item scale.

### Qualitative Findings

Three main categories along with 7 subcategories emerged from the analysis as shown in Table 4.

**Table 4.** Main categories and subcategories from the systematic text condensation analysis of focus group interviews.

Categories	Subcategories
Motivation for participation in the ASLEEP^a^ study	Engagement with society Interest in own sleep and sleep pattern
Benefits of the ASLEEP intervention	Increased knowledge of sleep and sleep patterns Increased awareness of sleep
Promoting and hindering factors of delivering a fully digitalized intervention	Flexibility User-friendliness Technical insecurity

a ASLEEP: Preventing and Treating Insomnia Symptoms in Mid-Life and Older Adults.

### Motivation for Participation in the ASLEEP Study

The participants were already engaged in research through their participation in PROTECT Norge and were invited to participate in the ASLEEP study through their involvement in PROTECT Norge. The motivation to join the ASLEEP study was 2-fold. Some participants joined the study as a form of civic duty or out of an interest in learning more about their own sleep to improve sleep.

#### Engagement With Society

Most participants stated an interest in contributing to research as part of their engagement with society, but for various reasons. A few participants talked about how their participation in research was a form of contributing to society and being their choice of volunteer work—their way of giving something back to the community.

*This is my way of doing volunteer work for our society*.

Similarly, some participants expressed the same interest in contributing to research; however, they were driven by having gained insight into the importance of research because they had experienced family members who developed chronic illness.

#### Interest in Own Sleep and Sleep Patterns

Some of the participants explained their interest in joining the study as wanting to learn more about sleep, showing interest in their own sleep and sleep patterns. Despite not explicitly stating that they had trouble sleeping per se, they expressed interest and curiosity tied to how their sleep “really was.”

*I think I sleep poorly, but I am not sure. I am curious to find out!*

Other participants had experienced specific sleep issues as they aged. For a few of the women, symptoms of insomnia had occurred as they entered menopause. Sleeping poorly was particularly challenging when having to combine poor sleep at night while simultaneously having to perform at work. After retirement, they were still experiencing sleep issues, although now it was not as stressful as when they were working full-time. Furthermore, several of the participants wanted to learn more about what they could do themselves to improve their sleep:

*I would like to learn more about what I can do myself in order to improve my sleep pattern*.

### Benefits of the ASLEEP Intervention

All participants, including those who did and did not explicitly express having poor sleep, reported experiencing beneficial outcomes from the intervention, which combined digital psychoeducation, completing a sleep diary, and receiving tailored sleep feedback based on sleep diary data through a sleep report. They expressed having learned something new and having made new realizations regarding their own sleep.

#### Increased Knowledge of Sleep and Sleep Patterns

Through the video modules in the intervention, they were given insight into the different phases of sleep. Even though knowledge about sleep stages, such as deep sleep and REM sleep, was common to some of the participants, they appreciated the systematic way it was presented. This was very useful in recognizing and understanding their own sleep pattern:

*It was very valuable to confirm my suspicion of my own sleep pattern, I wake up too many times during the night, and especially in the REM-sleep phase. This was actually easy for me to recognize from the explanation in the video.*

Several of the participants commented that the content of the videos was research-based, systematically presented by an expert in the field, and the dialogue made it interesting and, at the same time, easy to follow.

*I liked that the teaching in the video was in the form of an interview*.

Because the content of the videos was conveyed to them by an expert in the field of sleep, some participants expressed a feeling that both they and their sleep-related challenges were being taken seriously.

#### Increased Awareness of Sleep

Through the sleep diary, the participants were asked to make notes of their sleep every day. This meant that they had to reflect on their own sleep every day, which led to an increased awareness of sleep itself, as well as their own sleep. For some, this was a greater challenge than for others. One participant, who expressed experiencing symptoms of insomnia, had developed a coping strategy of deliberately avoiding thoughts about sleep. The participant had learned that by not thinking of how long they are awake, and similar concerns, it made the lack of sleep or sleep fragmentation less stressful:

*I had to do the opposite of what I am used to, instead of NOT thinking about how much I have slept - keeping a diary kind of “forced me” to do so. And I actually found this to be a good thing*.

Several of the participants also pointed out how being “forced” to reflect upon their own sleep patterns was a good thing:

*So what I found a bit positive and interesting was precisely that we are kind of forced to reflect on what happened during the night.*

Actually, most of the participants would have liked the diary to be more thorough, with more questions mapping their activities and habits that could affect their sleep patterns:

*I would have liked more documentation on what affects my sleep, in order to better my habits to improve my sleep.*

This proposed development of the sleep diary, based on what they would like to see in the sleep diary, was something that the participants brought up unsolicited during both groups.

### Promoting and Hindering Factors of Delivering a Fully Digitalized Intervention

The digitalized intervention was well-received and could be executed as planned, and minor technical issues were promptly resolved. The participants enjoyed the flexibility of being able to bring the iPad along with them, although some preferred their personal devices and would have liked to use those instead. Key hindering factors included technical insecurities, which varied among the participants but were mediated with technical support.

#### Flexibility

The fully digitalized intervention allowed the participants to complete it in their chosen location, enabling them to engage with and complete their study activities at a time and place of their choice. This flexibility was noted by all the participants as a very positive way of taking part in the study:

*I brought the iPad with me to the cabin, as I had planned to be there on holiday with my family, when the study took place.*

It was also possible for the participants to watch the videos at any time and frequently as they liked:

*It was nice to have the videos on the iPad, where I could pause and watch them again. I saw the last video three times, very interesting*.

#### User-Friendliness

Each participant was given an iPad dedicated to use in the study, which made the intervention easily accessible:

*It is nice to have everything I need in the dedicated iPad, I do not have to look through all my other apps to find it*.

However, others would have liked to have the app on their own smartphone, instead of having yet another device:

*Could we have downloaded the app on our mobile instead? It feels kind of old fashioned to have to use an iPad for this*.

#### Technical Insecurity

Even though the overall impression of the intervention was positive, some participants expressed insecurity related to the use of a fully digitalized intervention. The main concern for the participants was their choice of answers when completing their sleep diaries. They would have liked a clear confirmation that they had reported their sleep correctly during the submission process. They felt insecure whether the app was functioning correctly at all times and, thus, getting the information they sent in a timely manner:

*I was a bit worried when I had submitted my answers, had I done this correctly?*

One concrete issue affecting the use of the app was whether the internet connection was stable or not:

*My internet is normally stable, but I did experience that it fell out and then everything stopped.*

The participants who were not familiar with using an iPad expressed that it was positive they were able to contact technical support when the app was unstable or did not work correctly. However, there seemed to be a difference between those who were experienced iPad users, as they took matters into their own hands:

*I just exited the app and open the app again, like I usually do*.

## Discussion

This study explored participants’ experiences of engaging with the ASLEEP eHealth intervention. Participants’ motivations reflected both an altruistic desire to contribute to research and a personal curiosity about their own sleep. This mix of civic engagement and self-interest appeared to foster strong participation, even among individuals who did not initially report major sleep difficulties. Such engagement may reflect characteristics of the PROTECT Norge cohort, participants who are highly research-oriented and receptive to aging and brain-health studies [25], and is consistent with prior reports of strong public interest in brain-health initiatives [37]. The majority of participants were either curious to learn more about their own sleep or motivated to address ongoing sleep difficulties. These varied motivations can be understood through the lens of the transtheoretical model of behavior change, which conceptualizes behavior change as a progression through distinct stages, such as precontemplation, contemplation, preparation, action, maintenance, and relapse [38]. Within this framework, some participants’ curiosity about their sleep may reflect the contemplation stage, characterized by the awareness of a potential issue but limited action, while others who sought concrete strategies to improve sleep exemplify the preparation and action stages. This diversity highlights how ASLEEP engaged participants across motivational stages, although it is worth noting that the stages were not empirically measured here. Participants’ insomnia severity scores indicated a spectrum from nonclinically significant insomnia to subthreshold levels, suggesting that none were experiencing severe sleep disturbance. Nevertheless, 9 participants reported sleep problems persisting for at least 3 months, implying a mix of chronic but subclinical sleep complaints within the sample. Together, the results from this small feasibility and acceptability study indicate that the ASLEEP intervention may appeal to midlife and older adults with different self-described motivations for participating, particularly those with mild or emerging sleep difficulties and generally good mental health—aside from one case of moderate-to-severe depression.

Building eHealth interventions on established psychological behavior-change frameworks has been shown to enhance intervention efficacy. A systematic review of internet-based health programs found that theory-driven designs incorporating multiple behavior-change techniques and diverse communication modes achieved the strongest outcomes [18]. Guided by these principles, the ASLEEP intervention was developed with an educative focus, drawing on evidence-based components of CBT-I and psychoeducation. It was designed not only to address sleep-wake disturbances but also to promote broader sleep health in line with the Buysse [39] conceptualization. The educative approach adopted in ASLEEP was grounded in the health-promotion principle that greater knowledge—and the awareness it fosters—can lead to positive changes in attitudes and behavior [40]. Qualitative findings from this study suggest that ASLEEP participants experienced increased knowledge of sleep and sleep patterns and increased awareness of sleep, which, within a health-promotion framework, may be understood as an important first step toward addressing poor sleep and maintaining sleep health.

ASLEEP was perceived as valuable in enhancing participants’ understanding of sleep and increasing their awareness of personal sleep habits and patterns. The psychoeducational video modules—delivered through structured expert dialogue—were well received and sustained engagement even among participants already familiar with parts of the content. Gaining insight into normal sleep patterns appeared particularly helpful in correcting personal misconceptions about sleep, reflecting a key mechanism in CBT-I aimed at reducing sleep-related anxiety and promoting healthier sleep behaviors [11]. Self-monitoring through the daily sleep diary was also viewed as beneficial. Recording sleep patterns encouraged reflection, prompting participants to become more aware of their routines and identify opportunities for improvement. This process of increased self-awareness, developed through completing the week-long diary, aligns with previous research, showing that self-monitoring can promote greater awareness and adaptive changes in sleep behaviors [41,42]. Several participants suggested that the diary could be expanded to include items on sleep hygiene practices, believing that more targeted reflection would help them make better-informed decisions about daily habits. This view is consistent with prior findings in student populations that sleep diaries focusing on specific sleep hygiene behaviors are more effective in improving sleep outcomes [43]. This reflexivity also suggests a relatively high level of health literacy, defined as the ability to access, understand, and apply health information in daily life [44], a trait often associated with higher educational attainment [45]. This study sample was highly educated, with 9 of 10 participants holding university-level qualifications.

Participants valued the flexibility enabled by ASLEEP’s digital format. However, their varied views regarding the dedicated iPad highlighted an important implementation consideration: the need to balance standardized intervention delivery with flexibility for participants, as the iPad supported a consistent study setup and was preferred by some, while others would have preferred using their own devices for smoother integration into daily routines. Ensuring that eHealth tools accommodate individual preferences and patterns of technology use is central to maintaining engagement and effective use [17]. Expanding future versions of ASLEEP to be compatible across multiple platforms—especially smartphones—could therefore broaden accessibility and improve usability among the target group. A few participants expressed uncertainty about whether they were interacting with the application correctly, reflecting a sense of digital uncertainty. Such experiences illustrate a broader issue within digital health initiatives, where uneven digital competence and confidence can affect engagement [46]. At the same time, these individual challenges occur within a wider European context of accelerating digitalization and growing emphasis on digital participation [13]. In this study, we find readily available technical assistance through the PROTECT Norge helpdesk, which was important in alleviating participants’ digital uncertainty, particularly among those less accustomed to tablet-based technology. Ensuring that eHealth interventions provide accessible and responsive technical support is therefore essential for promoting confidence and equitable participation among middle-aged and older adults with varying levels of digital literacy [16,17].

The PROTECT Norge infrastructure played a central role in supporting rapid recruitment and efficient study coordination, which in turn contributed to a high level of participant compliance. By combining the consent for contact system with postal-code filtering, 508 eligible individuals were identified within the target area. Recruitment was completed within 5 days, during which 150 invitations were distributed, resulting in 18 expressions of interest and the inclusion of 10 participants. Participant engagement and data collection were also strong throughout the study period. Within the PROTECT platform, automated reminder functions and real-time monitoring of participant activity via a whitelist report enabled timely and personalized follow-up. These features likely contributed to the very high compliance rates observed—99.3% of sleep diary entries and full completion of baseline assessments. This illustrates the practical advantages of embedding nested studies such as ASLEEP within the established PROTECT Norge digital research platform, which effectively supports both recruitment and study activity adherence.

PPIE, through WiseAge and mainly through the study’s user representatives, helped ensure that study materials and procedures were clear, relevant, and accessible to participants. It provided practical values by incorporating structured user feedback during the preparation phase and enabled minor adjustments to be made before the feasibility test.

The transferability of these findings beyond the PROTECT Norge cohort may be limited by the sample’s distinctive characteristics, notably a strong intrinsic motivation to participate in aging and cognitive-health research and high educational attainment [25]. Higher educational attainment is often associated with greater health literacy [44,45], and because participation in an online study presupposes at least basic digital competence [13], the PROTECT Norge cohort likely represents a subgroup with high digital competence and confidence. Moreover, the use of a self-referral recruitment process in ASLEEP may have further amplified this profile, attracting particularly motivated and digitally confident participants.

The ASLEEP intervention was well-received, and participants reported a new understanding of sleep through educational videos, a sleep diary, and personalized feedback, primarily through increased knowledge and awareness. While some participants experienced technological insecurities, these were mitigated by the technical support provided. Improving app stability and expanding device compatibility could further enhance accessibility and optimize the eHealth intervention’s acceptability among individuals aged 50 years and over from the PROTECT Norge cohort. The PROTECT Norge infrastructure demonstrated good feasibility in recruitment, participant management, and maintaining high compliance, thus contributing to the timely and successful execution of the ASLEEP study as a nested study within PROTECT Norge.

## Supplementary material

10.2196/86591 Checklist 1 SRQR checklist.

## Abbreviations

ASLEEP: Preventing and Treating Insomnia Symptoms in Mid-Life and Older Adults
CBT-I: cognitive behavioral therapy for insomnia
eCBT-I: digitally delivered cognitive behavioral therapy for insomnia
GAD-7: Generalized Anxiety Disorder 7-item scale
GRIPP2: Guidance for Reporting Involvement of Patients and the Public, Version 2
PHQ-9: Patient Health Questionnaire-9
PPIE: patient and public involvement and engagement
PROTECT Norge: Platform for Research Online to Investigate Genetics and Cognition in Ageing Norge
REM: rapid eye movement
SESAM: Centre for Age-Related Medicine
STC: systematic text condensation
