# Supplementary material for: The ASLEEP Intervention for Insomnia Symptoms in Mid-Life and Older Adults supported by the PROTECT Norge Platform: Single-Arm, Multimethod Feasibility and Acceptability Study
Source: JMIR Form Res. 2026 Jul 30;10:e86591. doi: 10.2196/86591 (PMC13422585; doi:10.2196/86591)
Supplement: Checklist 1 [file formative-v10-e86591-s001.pdf]

The SRQR Reporting Checklist

Item	Description	Location in manuscript (or reason for not reporting)
**Title & Abstract**		
**Title**	Describe the nature and topic of the study. Identify the study as qualitative or indicate the approach or data collection methods.	The title does not explicitly identify the study as qualitative or specify focus group interviews because the title was prioritized to reflect the primary purpose of the study: assessing feasibility and acceptability of the ASLEEP intervention within the MRC framework for developing and evaluating complex interventions. The qualitative approach and focus group interviews are reported in the Abstract and Methods.
**Abstract**	Summarise the key elements of the study using the abstract format of the intended publication.	Abstract (p.1-2)
**Introduction**		
**Problem formulation**	Describe the problem/phenomenon studied, its significance, relevant theory and empirical work, and gaps in current knowledge.	Introduction (p. 2-3)
**Purpose or research question**	Describe the purpose of the study and specific objectives or questions.	Introduction (p. 3)
**Methods**		
**Qualitative approach and research paradigm**	Describe your qualitative approach, your guiding theory, if appropriate, and research paradigm, and reasons for your choices.	Methods, Study Design (p. 3);<br>Methods, Qualitative analysis (p. 9-10)
**Researcher characteristics and reflexivity**	Describe how researchers' characteristics may influence the research, including personal attributes, qualifications/experience, relationship with participants, assumptions, and/or presuppositions; potential or actual interaction between researchers' characteristics and the research questions, approach, methods, results and/or transferability.	Methods, Qualitative data collection (p. 6);<br>Methods, Qualitative analysis (p. 9);<br>Discussion (p.19)
**Context**	Describe the setting/site(s) in which the study was conducted, why it was selected, and any other salient contextual factors that may influence the study.	Methods, Intervention and Setting (p. 3-5);<br>Methods, Participants and Recruitment (p. 5-6)
**Sampling strategy**	Describe how and why research participants, documents, or events were selected; criteria for deciding when no further sampling was necessary, and the rationale for those criteria.	Methods, Participants and Recruitment (p.5) ;<br>Methods, Sample size (p. 6)
**Ethical issues pertaining to human subjects**	Describe any approval by an appropriate ethics review board and participant consent, or explain any lack thereof. Describe any other confidentiality and data security issues.	Methods; Ethical Considerations (p. 10)
**Data collection methods**	Describe the types of data collected; details of data collection procedures including, as appropriate, start and stop dates of data collection and analysis, iterative process, triangulation of sources/methods, and modification of procedures in response to evolving study findings. Describe your rationale for these choices.	Methods, Setting (p. 4-5);<br>Methods, Recruitment process (p. 5);<br>Methods, Data collection (p. 6-7);<br>Figure 1 (p. 9)
**Data collection instruments and technologies**	Describe any instruments, e.g., interview guides, questionnaires, and devices, e.g., audio recorders, used for data collection; describe if/how the instrument(s) changed over the course of the study.	Methods, Intervention (p. 3-4);<br>Methods, Quantitative data collection (p. 6);<br>Methods, Qualitative data collection, incl.Table 2 (pp. 6-9);<br>Figure 1 (p. 9)

Item	Description	Location in manuscript (or reason for not reporting)
**Units of study**	Describe the number and relevant characteristics of participants, documents, or events included in the study. Describe the level of participation.	Methods, Qualitative data collection (p. 6);<br>Methods, Qualitative analysis (p. 9);<br>Methods, Ethical Considerations (p. 10);<br>Discussion, Data Availability (p. 19)
**Data processing**	Describe the methods for processing data prior to and during analysis, including transcription, data entry, data management and security, verification of data integrity, data coding, and anonymisation/deidentification of excerpts.	Methods, Qualitative data collection (p. 6);<br>Methods, Qualitative analysis (p. 9);<br>Methods, Ethical Considerations (p. 10);<br>Discussion, Data Availability (p. 19)
**Data analysis**	Describe the process by which inferences, themes, etc. were identified and developed, including the researchers involved in data analysis; usually references a specific paradigm or approach. Describe why you chose this process.	Methods, Qualitative analysis (pp. 9-10)
**Techniques to enhance trustworthiness**	Describe any techniques to enhance trustworthiness and credibility of data analysis, e.g., member checking, triangulation, audit trail. Describe why you chose these techniques.	Methods; Qualitative data collection (p. 6);<br>Methods, Qualitative analysis (pp. 9-10);
**Results**		
**Synthesis and interpretation**	Describe the main findings, e.g., interpretations, inferences, and themes; might include development of a theory or model, or integration with prior research or theory.	Results; Qualitative findings (p. 13-16);<br>Table 5 (p. 13)
**Links to empirical data**	Provide evidence, e.g., quotes, field notes, text excerpts, photographs, to substantiate analytic findings.	Results; Qualitative findings (p. 13-16)
**Discussion**		
**Integration with prior work, implications, transferability, and contribution(s) to the field**	Summarize the main findings; explain how findings and conclusions connect to, support, elaborate on, or challenge conclusions of earlier scholarship; discuss the scope of application/generalizability; identify unique contribution(s) to scholarship in a discipline or field.	Discussion (p. 16-19);
**Limitations**	Discuss the trustworthiness and limitations of findings.	Discussion, (p. 19)
**Other**		
**Conflicts of interest**	Describe any potential sources of influence or perceived influence on study conduct and conclusions. Describe how these were managed.	Conflict of Interest (p. 19)
**Funding**	Describe sources of funding and other support. Describe the role of funders in data collection, interpretation, and reporting.	Funding (p. 19)

Citation note: O'Brien BC, Harris IB, Beckman TJ, Reed DA, Cook DA. Standards for reporting qualitative research: A synthesis of recommendations. Academic Medicine. 2014;89(9):1245–1251.

Følsomhet Intern (gul)
